# Measuring socioeconomic inequalities in postnatal health checks for newborns in Ethiopia: a decomposition analysis

**DOI:** 10.3389/fpubh.2024.1384729

**Published:** 2024-06-05

**Authors:** Asebe Hagos, Misganaw Guadie Tiruneh, Kaleab Mesfin Abera, Yawkal Tsega, Abel Endawkie, Wubshet Debebe Negash, Amare Mesfin Workie, Lamrot Yohannes, Mihret Getnet, Nigusu Worku, Adina Yeshambel Belay, Lakew Asmare, Hiwot Tadesse Alemu, Demiss Mulatu Geberu, Kaleb Assegid Demissie, Melak Jejaw

**Affiliations:** ^1^Department of Health Systems and Policy, Institute of Public Health, College of Medicine and Health Science, University of Gondar, Gondar, Ethiopia; ^2^Department of Health Policy and Systems, Institute of Public Health, College of Medicine and Health Science, Wollo University, Dessie, Ethiopia; ^3^Department of Health System and Management, School of Public Health, College of Medicine and Health Sciences, Wollo University, Dessie, Ethiopia; ^4^Department of Epidemiology and Biostatistics, School of Public Health, College of Medicine and Health Sciences, Wollo University, Dessie, Ethiopia; ^5^Department of Nutrition and Dietetics, Institute of Public Health, College of Medicine and Health Science, University of Gondar, Gondar, Ethiopia; ^6^Department of Environmental and Occupational Health and Safety, Institute of Public Health, College of Medicine and Health Science, University of Gondar, Gondar, Ethiopia; ^7^Department of Human Physiology, School of Medicine, College of Medicine and Health Sciences, University of Gondar, Gondar, Ethiopia; ^8^Department of Epidemiology and Biostatistics, Institute of Public Health, College of Medicine and Health Sciences, University of Gondar, Gondar, Ethiopia

**Keywords:** inequities, socioeconomic inequality, health checkup, newborn, concentration curve, concentration index, decomposition analysis, Ethiopia

## Abstract

**Background:**

Addressing health inequity is a top priority for achieving sustainable development goals. The existing evidences in Ethiopia have shown that there are substantial inequalities in the use of health services among various socioeconomic strata. Therefore, the present study aimed to measure socioeconomic inequalities and the contributing factors in postnatal health checks for newborns in Ethiopia.

**Methods:**

We used a secondary data from the recent 2019 Ethiopia Mini Demographic and Health Survey dataset. The study includes a weighted sample of 2,105 women who gave birth in the 2 years preceding to the survey. The study participants were selected using two stage cluster sampling techniques. The socioeconomic inequality in postnatal health checks for newborns was measured using the Erreygers Normalized Concentration Index (ECI) and illustrated by the concentration curve. A decomposition analysis was done to identify factors contributing to the socioeconomic related inequality in postnatal health checks for newborns in Ethiopia.

**Results:**

The concentration curve of postnatal health checks for newborns lay below the line of equality, and the Erreygers normalized concentration index was 0.133, with a standard error = 0.0333, and a *p* value <0.001; indicating that the postnatal health check for newborns was disproportionately concentrated among newborns with higher socioeconomic status. The decomposition analysis reported that antenatal care (ANC) visit (59.22%), household wealth index (34.43%), and educational level of the mother (8.58%) were the major contributors to the pro-rich socioeconomic inequalities in postnatal health checks for newborns.

**Conclusion:**

The finding revealed that there is a pro-rich inequality in postnatal health checks for newborns in Ethiopia. To reduce the observed socioeconomic health inequality, the government needs to improve ANC visits, implement strategies to access health service for economically disadvantaged groups, and increase educational attainment among women.

## Background

Globally, there has been a substantial improvement in child survival and newborn health during the last 30 years. The number of neonatal deaths fell from 5 million to 2.3 million between 1990 and 2021, and the neonatal mortality rate was reduced by more than half, from 37 to 18 deaths per 1,000 live births. In 2019, approximately 6,700 neonatal deaths occur each day, accounting for 47% of all child mortality under the age of five (1–3). According to 2019 world health organization (WHO) report, neonatal mortality rates (NMRs) vary greatly throughout the world; from 0.9 in Japan, to 44 deaths per 1,000 live births in Pakistan and nearly 75% of newborn deaths occur in Southern Asia and sub-Saharan Africa (4, 5). Globally, in 2020, five countries accounted for more than half of all newborn deaths, which means the number of newborn deaths was reported at 468,000 in India; 277,000 in Nigeria, 257,000 in Pakistan, 106,000 in the Democratic Republic of the Congo, and 104,000 in Ethiopia (2).

According to the 2019 Ethiopia Minin Demographic Health Survey (EMDHS), NMR increased in Ethiopia from 29/1000 in 2016 to 33/1000 in 2019 (6). Moreover, greater disparities in NMR were observed among regions, with particularly high mortality rates in the Benishangul-Gumuz and Somali regions (7). The disparities in NMR were much more increased on the basis of educational level, residence, and wealth status (7, 8).

Studies on neonatal mortality report that about half of newborn deaths occur within the first 2 days of life (3, 9). Such evidence suggests newborns are most vulnerable during the first hours and days of life. Postnatal health checks for newborns within 2 days after birth are very important to provide essential newborn care and to detect any neonatal complications early (1, 6). Studies in Ethiopia showed that the proportion of newborns receiving adequate content of postnatal health checks ranged from 8 to 16% (10–13). Additionally, another study reported that 84.3% of newborns did not receive postnatal health checks within 2 days after birth (14). Based on the 2019 EMDHS report, within the past 20 years, Ethiopia has made impressive progress on a number of maternal and child health indicators. However, the socioeconomic inequalities in health service coverage and utilization have persisted over the past two decades between rural and urban populations, as well as between the wealthiest and poorest (6, 15).

Health outcomes are significantly influenced by social, economic, and environmental factors. Disparities in health outcomes are caused by these factors, which are typically distributed unevenly across socioeconomic groups (16). Currently, health inequality is a global concern and has become a health policy goal in the health system (17). Health inequalities are observable differences in health between subgroups of a population. Subgroups can be defined by demographic, geographic, or socioeconomic factors such as age, economic status, education, place of residence, and sex (18).

Every child has the right to survive, according to the United Nations Convention on the Rights of the Child. This comprises the most vulnerable newborns residing in disadvantaged communities. From the standpoint of public health, the most vulnerable newborns are those who are born into marginalized communities, such as rural residents, urban slums, and those with poor socioeconomic status, who are most likely to die (4). Studies have reported that there has been a growing concern related to inequalities in maternal health service utilization and postnatal health checks and newborn in various regions of the world (19).

Currently, the utilization of postnatal health care services is not fairly distributed in low and middle income countries, and it varies significantly on the basis of socioeconomic status (20). In Ethiopia, studies have described the existence of substantial inequality in the utilization of health services including postnatal health checks across socioeconomic groups (7, 12, 21). Findings from southwest Ethiopia highlighted that neonatal health service utilization was highly concentrated among newborns from educated mothers and households (21). Likewise, another research also found that the quality of postnatal newborn care is more prevalent among newborns from higher socioeconomic households (12).

The government of Ethiopia has developed a national health equity strategic plan to address the issue of inequality in health and ensure that no one is left behind. Consequently, having recent data on the level of inequality is vital to enhance effort toward narrowing the inequality across socioeconomic strata. However, the level of socioeconomic related health inequality in postnatal health checks for newborns and the contributing factors for the socioeconomic inequalities have not been well investigated in Ethiopia. Furthermore, to the best of our knowledge, no studies have been conducted to measure socioeconomic inequality in postnatal health checks for newborns in Ethiopia using the 2019 EMDHS data.

Therefore, this study aimed to measure socioeconomic inequalities and the contributing factors in postnatal health checks for newborns in Ethiopia. The findings of this study can help policy makers and health managers to formulate evidence-driven approaches to reduce socioeconomic inequality in postnatal health checks for newborns in Ethiopia.

## Methods

### Study setting and period

Currently, Ethiopia has 12 administrative regions and two city administrations. Ethiopia’s population is estimated to be 126 million people, making it the second most populous country in Africa (22). For this study, we used data from the 2019 EMDHS dataset. EMDHS is a nationally representative household survey and was conducted from March 21 to June 28, 2019. The survey was carried out to provide updated information on selected maternal and child health outcomes.

### Study population, sample size, and sampling procedure

**Figure 1:**
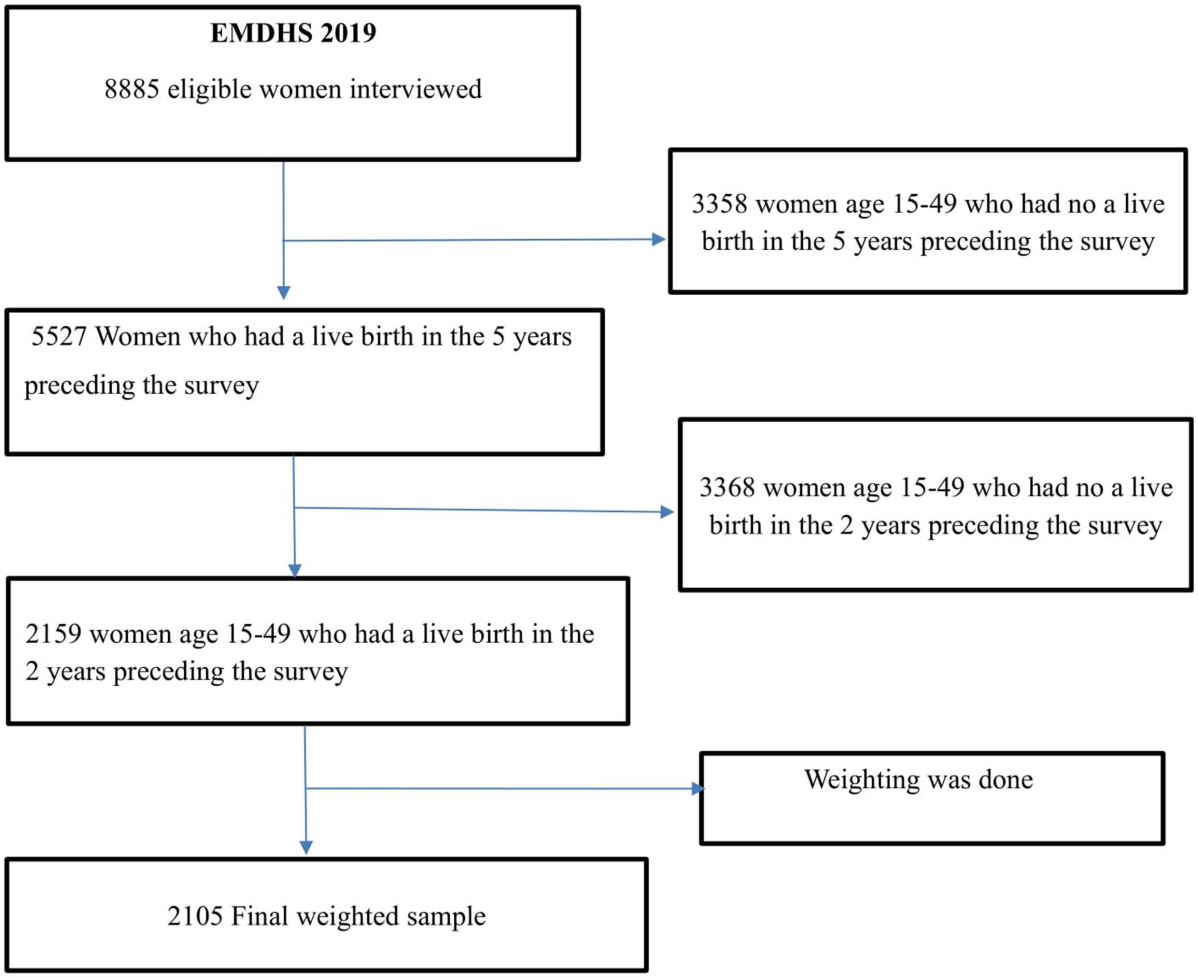
The final sample size and a schematic presentation of how the study sample was selected.

Women between the ages of 15 and 49 who had given birth in the 2 years before the survey in the selected enumeration areas (EAs) were the study population. The study included 2,105 weighted samples (Figure 1).

Using a two-stage stratified cluster sampling technique, each region was divided into urban and rural areas. Accordingly, 21 sampling strata were created, and an equal allocation of samples was done in each region, wherein 25 EAs were selected from eight regions, whereas, 35 EAs were selected from three larger regions.

In the first stage, 305 clusters (93 urban and 212 rural) were selected with probability proportional to EAs size and with independent selection in each sampling stratum. In the second stage, a fixed number of 30 households per cluster was selected. Finally, women aged 15–49 in 9,150 (2,790 urban and 6,360 rural) households from 305 clusters were selected. Detail information about the whole procedure of sampling is available on the 2019 EMDHS report (6).

### Study variables

In this study, the outcome variable was postnatal health checks for newborns within 2 days after birth. It was created by computing the five contents of postnatal care for newborns. The computing variables were cord examined, temperature measured, counseling on danger signs, counseling on breastfeeding, and observation of breastfeeding. The postnatal health check for newborns was coded as 1 “yes” if a woman had received the five selected contents of postnatal care for her baby within 2 days, otherwise coded as 0 “no” if the woman had not received the five selected contents of postnatal care (12–14, 23, 24).

The potential contributing factors considered for the decomposition analysis were identified through reviewing of various literatures sources (10, 11, 25–27). Place of residence (urban and rural), age of the mother (15–19, 20–34, and 35–49 years), education level of the mother (no education, primary education, secondary and higher education), respondent’s religion (Orthodox, Protestant, Muslim, others), marital status of the mother (single and married), sex of the household head (male and female), administrative regions of Ethiopia (city administrations, established regions, and emerging regions), antenatal care visits (no ANC visits, 1–3 ANC visits, and 4 and above ANC visits), and birth order of the child (first, second or third, fourth or fifth, and sixth or above).

During the survey period the country had nine regions and two city administrations. However, this study classified the regions into three groups according to their respective levels of economic growth and infrastructure accessibility. The established regions are relatively developed and include Tigray, Amhara, Oromia, Southern Nation Nationalities and People (SNNP), and Harari; the emerging regions are relatively less developed and include Afar, Somali, Benishangul-Gumuz, and Gambela, which are primarily pastoral people. Addis Ababa and Dire Dawa are considered urban areas (25, 26).

### Measurement of socioeconomic status

The DHS surveys do not collect information on income or expenditure, wealth index is frequently used in DHS as a relative measure of the socioeconomic status of households in low- and middle-income countries (27, 28).

The wealth index is constructed from several indicators that are assumed to be connected with a household’s wealth status. The indicators include, for example, possession of assets such as electricity, television, radio, watch, telephone, refrigerator, types of vehicles, and variables describing the water supply and dwelling such as the source of drinking water, type of toilet, material of principal floor, walls, roof, cooking fuel, and ownership of agricultural land, type and number of animals owned (29, 30). Principal component analysis was used to construct the wealth index of the households (31). The wealth index of the household ranked in five quantiles (poorest, poorer, middle, wealthier, and wealthiest).

### Statistical analysis

#### Measuring inequality in postnatal health check for newborns

Socioeconomic inequalities of postnatal health check for newborns were measured using the concentration curve (CC) and concentration index (CI). The CC plots the cumulative proportion of the postnatal health check on newborns in the y-axis against the cumulative proportion of the population ranked by wealth index, ranked from the poorest to the richest, in the x-axis. If the postnatal health check for newborns is equally distributed across the socioeconomic groups, the concentration curve will coincide with the diagonal line (line of equality). If the postnatal health check for newborns is concentrated in the higher socioeconomic groups, the concentration curve will lie below the diagonal line (32, 33).

The concentration curve measures the socioeconomic inequality of health care utilization across socioeconomic groups. However, it does not provide a measure of the magnitude of inequality. The concentration index is defined as twice the area between the concentration curve and the line of equality (the 45-degree line). It provides a measure of the extent of inequalities in postnatal health checks for newborns. Thus, we used the concentration index to measure the extent (degree) of wealth-related inequality of postnatal health checks for newborn (32–35).

Since the outcome variable is binary, we used Erreygers normalized concentration index, as shown in Equation 1 (ECI) (36).


1 $$ECI=4*\mu *CI*\left(y\right)$$

Where ECI is the Erreygers concentration index, μ is the mean of the postnatal health check for newborns and CI is the generalized concentration index. The value of ECI ranges from −1 to +1. When the value of ECI is positive, it indicates the postnatal health check for newborns is disproportionately concentrated among the rich (pro rich). If it is negative, it indicates the postnatal health checks for newborns is disproportionately concentrated among the poor (pro poor). Larger absolute values of CI indicate wider inequalities in postnatal health checks for newborns (32, 37). When an index is 0, it shows an absence of socioeconomic inequalities in postnatal health checks for newborns.

### Decomposition analysis

Identifying the cause of inequality is a critical step in addressing socioeconomic inequality in health. Due to this reason, policymakers and researchers are concerned in investigating the contributing factors of socioeconomic inequality in health (38, 39). Therefore, a decomposition analysis of ECI was done to identify factors contributing to socioeconomic related inequality in the postnatal health check of newborns.

Equation 2 shows that for any linear additive regression model of the health outcome variable (32), y, such as


2 $$y=\alpha +\underset{\dot{k}}{\sum }\beta_{k}x_{k}+\varepsilon$$

The concentration index for y, CI, is given as:


3 $$CI=\underset{k}{\sum }\left(\frac{\beta_{k}{\bar{x}}_{k}}{\mu }\right)CI_{k}+\frac{GC\varepsilon }{\mu }$$

Where “y” is the health outcome variable (in this case postnatal health check for newborn), $x_{k}$is a set of the socioeconomic determinants of the outcome variable, α is intercept, and $\beta_{k}$ is the coefficient of $x_{k}$and, ε is the residual of the error term.

Where “CI” represents the overall concentration index, μ indicates the mean of y (health outcome variable), ${\bar{x}}_{k}$ represents the mean of $x_{k}$ (determinants), $CI_{k}$ is the concentration index for $x_{k}$, and$GC_{\varepsilon }$represents the generalized concentration index for ε.

Equation 3 shows that the overall inequality in health outcomes has two components: an explained component and an unexplained component; the explained component $(\underset{k}{\sum }\left(\frac{\beta_{k}{\bar{x}}_{k}}{\mu }\right)CI_{k}$), which indicates the contribution of each explanatory variable to the socioeconomic inequality of postnatal health checks for newborns, and the unexplained (residual) component ($\frac{GC\varepsilon }{\mu }$), indicates the socioeconomic inequality of postnatal health check for newborn that cannot be explained by systematic variations across income groups in the $x_{k}$,which should be approach zero for a well specified model (32).

The decomposition approach was initially introduced to use with a linear, additively separable model. However, the outcome variable, the postnatal health check for newborn is non-linear. Thus, a probit regression model is applied to analyze the influences of determinants on the probability of a postnatal health check for newborns. One possibility when dealing with a discrete change from 0 to 1 is to use marginal or partial effects, which show the change in an explanatory variable (40–42). The marginal or partial effects have been analyzed in the analysis of health sector inequalities in non-linear settings (40, 43).

Equation 4 explains the linear approximation of the non-linear estimations


4 $$CI=\underset{k}{\sum }\left(\frac{\beta_{\dot{k}}^{m},{\bar{x}}_{k}}{\mu }\right)CI_{k}+\frac{GC\varepsilon }{\mu }$$

Where $\beta_{\dot{k}}^{m}$, is the marginal effect of the explanatory variable $x_{k}$(dy/dxk).

The procedure in decomposition analysis can be summarized in the following steps: The first, the regression model of the health outcome variable is performed for all ${\bar{x}}_{k}$ to obtain the marginal effects of determinants $\beta_{\dot{k}}^{m}$, which show the association between the explanatory variable and the outcome variable (postnatal health check for newborn). In the second step, the elasticity of the outcome variables was estimated for each x (${\bar{x}}_{k}$), which is a level of responsiveness of the outcome variable to a change in its determinant variable $\left(\frac{\beta_{\dot{k}}^{m},{\bar{x}}_{k}}{\mu }\right)$. In the third step, the CI is calculated for the health outcome variable and each explanatory variable. In the fourth step, the contribution of each explanatory variable to the overall ECI is calculated by multiplying the elasticity of each determinant by its concentration index $\left(\frac{\beta_{\dot{k}}^{m},{\bar{x}}_{k}}{\mu }\right)CI_{k}$. Finally, the percentage contribution of each explanatory variable to the overall inequality was obtained by dividing its contribution by the ECI and multiplying by one hundred (26, 32, 43–45). Stata version 17 was used to carry out the statistical analysis.

## Results

### Background characteristics of the study participants

A total of 2,105 women who gave birth in the 2 years preceding the survey were participated in the present study. Nearly, three-fourth 1,538 (73.06%) of the women were 20–34 years old. The vast majority of the women 1982 (94.1%) were married. Nearly, half 978 (46.4%) of respondents were had no education. The majority of the study participants were from rural areas 1,552 (73.7%) and the established regions 1830 (86.9%). Additionally, 926 (43.9%) of women had four and above ANC visits, whereas, 550 (26.13%) women had no any ANC follow-up (Table 1).

**Table 1:** Study participants characteristics and percentage of selected content of the postnatal health checks for newborn in Ethiopia (*N* = 2,105; EMDHS, 2019).

Characteristics	Category	Study participants		Received five selected contents of postnatal health checks for newborns	
		Frequency	Percent	Frequency	Percent
Age of the mother	15–19	180	8.55	9	5.00
	20–34	1,538	73.06	179	11.63
	35–49	387	18.39	47	12.14
Education level of the mother	No education	978	46.46	78	7.97
	Primary education	840	39.9	75	8.928
	Secondary or higher education	287	13.64	82	28.57
Respondent’s religion	Muslim	758	36.01	91	12.00
	Orthodox	753	35.77	108	14.34
	Protestant	555	26.37	35	6.30
	Others	39	1.85	1	2.56
Marital status of the mother	Single	123	5.85	12	9.75
	Married	1982	94.15	223	11.25
Sex of the household head	Male	1824	86.65	203	11.12
	Female	281	13.35	32	11.39
Place of residence	Urban	553	26.27	96	17.35
	Rural	1,552	73.73	139	8.95
Administrative regions of Ethiopia	Established regions	1830	86.94	183	10.00
	City administrations	77	3.66	33	42.85
	Emerging regions	198	9.4	19	9.59
Antenatal care visits	No ANC visits	550	26.13	5	0.90
	1–3 ANC visits	629	29.88	62	9.85
	4 and above ANC visits	926	43.99	168	18.14
Birth order of the child	First	500	23.76	57	11.4
	Second or third	721	34.25	97	13.45
	Fourth or fifth	446	21.19	54	12.10
	Sixth or above	438	20.8	27	6.16
Wealth index of the household	Poorest	460	21.85	26	5.65
	Poorer	450	21.37	33	7.33
	Middle	392	18.62	32	8.16
	Wealthier	364	17.3	35	9.61
	Wealthiest	439	20.86	109	24.82

### The prevalence of postnatal health check for newborns

The overall prevalence of selected content of postnatal health check for newborns within 2 days after birth in Ethiopia was 11.16 (95% CI: 8.6–14.2). The prevalence of postnatal health checks for newborn for urban residents was 17.3%, that is higher than that of rural residents 8.9%. Post natal health checks for newborn were 42.8% in city administration of Ethiopia, whereas, it was 10 and 9.5% in established and emerging regions of Ethiopia, respectively. Moreover, the prevalence of postnatal health checks for newborn was reported from respondents who attained secondary and above education 28.5%, and respondents from higher household wealth index (wealthiest) 24%, however the prevalence of postnatal health check for newborn from poorest household was 5.6%. Furthermore, women who had four and above ANC visits the prevalence of postnatal health checks for newborns was 18.1% (Table 1).

### Socioeconomic inequality in postnatal health checks for newborns

**Figure 2:**
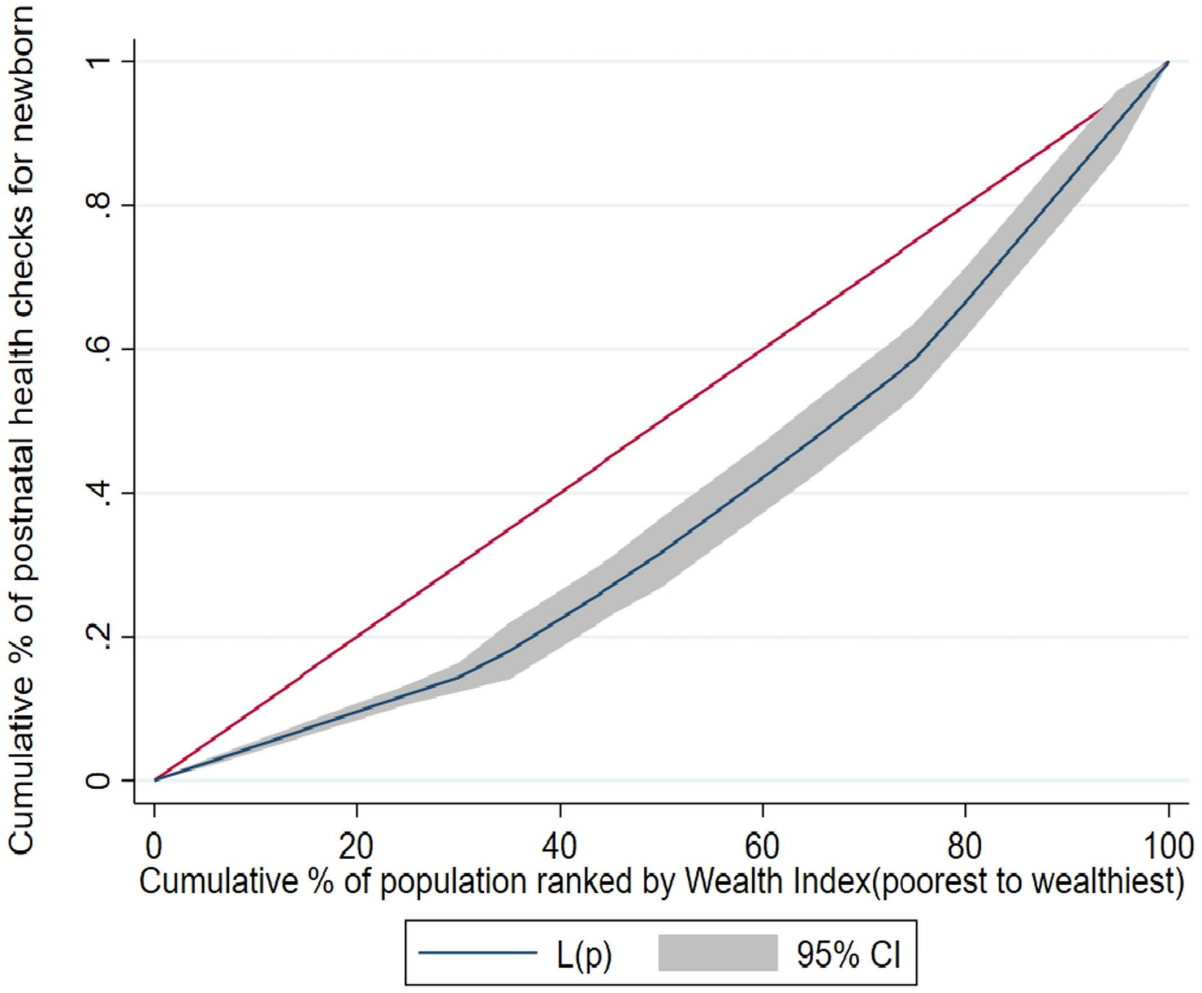
The concentration curve for postnatal health check for newborn in Ethiopia, 2019 EMDHS.

Figure 2 depicts the concentration curve of the postnatal health check for newborns in Ethiopia. The concentration curves lay below the 45° line, demonstrating that the postnatal health check for newborns was more concentrated amongst the wealthiest or higher socioeconomic groups (pro-rich distribution).

The overall ECI analysis of socioeconomic related inequality of postnatal checks for newborns in Ethiopia was 0.1336, with a standard error = 0.0333, *p* value <0.001. The positive sign of concentration indices in postnatal health checks for newborns confirms the presence of pro-rich inequalities. This concentration index, 0.1336 implies that the postnatal health checks for newborns is 13.36% higher among the rich or higher socioeconomic groups.

### Decomposing socioeconomic inequality in postnatal health checks for newborns

Findings from the decomposition analysis indicate the contributions of each factor to the overall socioeconomic inequality of the postnatal health check for newborns in Ethiopia. The marginal effect column from Table 2 indicated that variables such as secondary and higher education status, administrative regions of Ethiopia, antenatal care visits, and wealth index had a significant effect on the postnatal health check for newborns. Women from the city administration of Ethiopia had a 7% higher chance of receiving a postnatal health check for the newborn compared to those from established regions of Ethiopia. Similarly, women from wealthier households had a 9% higher probability of receiving a postnatal health check for the newborn when compared with those from the poorest. In contrast, compared to women who had four or more ANC visits, the likelihood of a postnatal health check for a newborn was 15% lower for women who had no ANC visits.

**Table 2:** A decomposition analysis of socioeconomic inequalities in the postnatal health check for newborn care in Ethiopia.

Characteristics		Marginal effects	Elasticity	Concentration index (ECI)	Absolute contribution	Percentage contribution	Summed %
Age of the mother	15–19	Base	Base	Base	Base	Base	1.23
	20–34	−0.0243	0.1135	0.1116	0.0046	3.4482	
	35–49	0.0005	0.0388	−0.0758	−0.0029	−2.2082	
Education level of the mother	No education	Base	Base	Base	Base	Base	8.58
	Primary education	0.0295	−0.0170	0.1117	−0.0019	−1.4266	
	Secondary and higher	0.0979^***^	0.0428	0.3119	0.0133	10.0119	
Respondent’s religion	Muslim	Base	Base	Base	Base	Base	−4.10
	Orthodox	0.0143	−0.0224	0.1697	−0.0038	−2.8547	
	Protestant	−0.0323	−0.0603	0.0333	−0.0020	−1.5072	
	Others	0.0200	−0.0162	−0.0213	0.0003	0.2591	
Marital status of the mother	Married	Base	Base	Base	Base	Base	0.02
	Single	0.0063	0.0042	0.0091	0.0000	0.0293	
Sex of the household head	Male	Base	Base	Base	Base	Base	−0.10
	Female	0.0331	−0.0096	0.0141	−0.0001	−0.1018	
Place of residence	Rural	Base	Base	Base	Base	Base	−19.58
	Urban	0.0242	−0.0516	0.5067	−0.0261	−19.5859	
Administrative regions of Ethiopia	Established regions	Base	Base	Base	Base	Base	−1.34
	City administrations	0.0733^**^	0.0157	0.1044	0.0016	1.2329	
	Emerging regions	0.0557^***^ 0.07830^***^	0.0209	−0.1642	−0.0034	−2.5785	
Antenatal care visits	4 and above ANC visits	Base	Base	Base	Base	Base	59.22
	1–3 ANC visits	−0.0483^***^	−0.0380	−0.0168	0.0006	0.4790	
	No ANC visits	−0.1495^***^	−0.2128	−0.3687	0.0784	58.7398	
Birth order of the child	First	0–0.0461	0.0041	0.1710	0.0007	0.5359	1.72
	Second or third	−0.0363	0.0494	0.1039	0.0051	3.8485	
	Fourth or fifth	−0.0052	0.0420	−0.0845	−0.0035	−2.6649	
	Sixth or above	Base	Base	Base	Base	Base	
Wealth index of the household	Poorest	Base	Base	Base	Base	Base	34.43
	Poorer	0.0660^*^	0.0005	−0.2983	−0.0001	−0.1318	
	Middle	0.0897^**^	0.0036	0.0379	0.0001	0.1036	
	Wealthier	0.0888^**^	0.0038	0.2839	0.0010	0.8143	
	Wealthiest	0.0626	0.0671	0.6597	0.0442	33.6452	
Explained							80.08
Residual							19.92

Significance level ***p-value < 0.001, **p-value < 0.01, *p-value < 0.05.

Elasticity is the sensitivity of the outcome variable to each explanatory variable. Table 2, elasticity column shows the elasticity of the postnatal health check for newborns. For instance, Women who reside in urban areas have an elasticity value of −0.0516, meaning that a change in residence from a rural to an urban area will reduce pro-rich socioeconomic inequality in postnatal health check in newborns by 5.1%. Conversely, the elasticity of household wealth index was found to be 0.0671, meaning that a change in women’s household wealth status from the poorest to the wealthiest would result in a 6.7% increment in the pro-rich socioeconomic inequality of postnatal health checks for newborns.

The concentration index in each variable describes the extent and direction of socioeconomic related inequality in postnatal health checks for newborns that corresponds to specific explanatory variables. Table 2, the ECI column show how each explanatory variable is distributed across socioeconomic status. Explanatory variable such as primary educational level (ECI = 0.1117), secondary and higher educational level (ECI = 0.3119), age of the mother 20–34 (ECI = 0.1116), being Orthodox religion follower (ECI = 0.1697), being Protestant religion follower (ECI = 0.0333), single marital status (ECI = 0.0091), male sex of the household head (ECI = 0.0141), urban resident (ECI = 0.5067), City administrations regions of Ethiopia (ECI = 0.1044), first birth order (ECI = 0.1710), second or third birth order (ECI = 0.1039), household wealth index middle (ECI = 0.0379), household wealth index wealthier (ECI = 0.2839), and household wealth index wealthiest (ECI = 0.6597) had positive Erreygers concentration index indicating that these variables were concentrated more among women with better socioeconomic status. In contrast, the age of the mother 35–49 (ECI = −0.0758), other religion follower (ECI = −0.0213), emerging regions of Ethiopia (ECI = –0.1642), no ANC visits (ECI = −0.3687), 1–3 ANC visits (ECI = −0.0168), fourth or fifth birth order (ECI = −0.0845), and poorer household wealth index (ECI = −0.2983) had a negative Erreygers concentration index, indicating these factors were more concentrated among economically disadvantaged women. This, therefore, implies that women from lower socioeconomic status were less likely to receive postnatal health check of newborn.

In this study, the contribution of each variable to the overall inequality of postnatal health checks for newborns was estimated and presented in Table 2. The explanatory variables included in our model explained 80.08% of the overall variability of socioeconomic inequality in postnatal health checks for newborns in Ethiopia. Antenatal care visits (59.22%), household wealth index (34.43%), and education level of the mother (8.58%) were the major contributors to the socioeconomic inequality in postnatal health checks for newborns. Birth order (1.72%) and age of mother (1.23%) had relatively minimal contribution to the inequality. On the contrary, women’s place of residence negatively contributed to inequality by −19.58%. About 19.92% of the overall inequality was contributed by unexplained variables or residuals.

## Discussion

The findings from concentration indices and decomposition analysis play a key role in informing evidence-based policy decisions, helping to reduce socioeconomic related health inequality (38, 46, 47). Therefore, study aimed to measure the level of socioeconomic inequalities and the contributing factors in postnatal health checks for newborns in Ethiopia. The result of this study demonstrated that there is socioeconomic inequality in postnatal health checks for newborns in Ethiopia. The findings of the present study imply that the postnatal health check for newborns was concentrated among newborns with high socioeconomic status. Our study is comparable with the findings of the previous studies (12, 20, 21). However, the magnitude of pro-rich inequalities in postnatal health checks for newborns was not comparable with the previous studies. In this study the magnitude of pro-rich inequalities was higher than in Vietnam (20). The difference might be explained by a variation in economic development, social policy, and the context of the health system. However, it was lower than the previous study in Ethiopia (12). The difference might be the fact that there was a difference in the study period and there was a variation in the measurement of the outcome variable. Several studies documented that women with higher socioeconomic status were more likely to use various maternal health services, such as ANC visits, skilled birth attendance, and postnatal health checks (15, 48–52). Newborns who belong to socioeconomically disadvantaged households experience a higher rate of mortality and morbidity. They are being left behind in accessing health services, in spite of having greater levels of health care need (32). In alignment with SDG 3.2, Ethiopia targets reducing the death of neonates to less than 12 per 1,000 live births in 2030. However, socioeconomic inequality in health can impose considerable challenge to attain the sustainable development goal of ending preventable newborns deaths. Thus, targeting reducing socioeconomic inequalities and ensuring the poorest have access to and utilize maternal and newborn services is essential to achieving universal coverage of maternal and neonatal healthcare (7).

The identification of contributing factors to the inequality in postnatal health checks for newborns is a critical step in designing relevant policy interventions to reduce the observed socioeconomic inequalities (38, 53). Accordingly, the decomposition analysis of this study identified various significant contributors to socioeconomic inequality in postnatal health checks for newborns. ANC visits, the wealth status of the household, and educational status of the mother were the factors with the highest contribution to the overall inequality of postnatal health checks for newborns.

**Figure 3:**
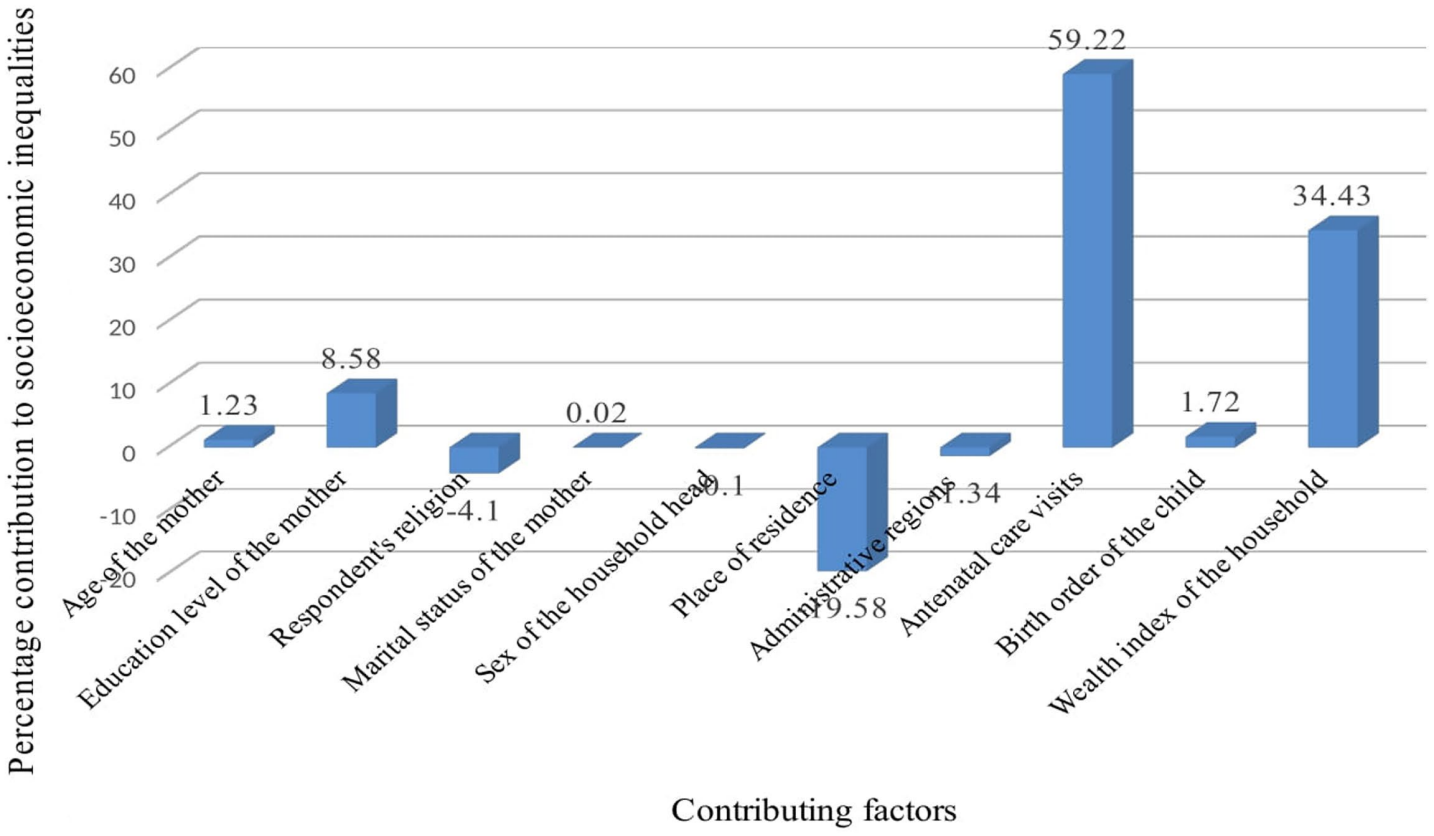
Contributions of various factors to postnatal health checks for newborns in Ethiopia.

In this study, ANC visit was a predominant contributor to the overall socioeconomic related health inequality (Figure 3). It explained about 59.22% of the total inequality in postnatal health checks for newborns. Existing studies have documented that ANC visit is an important contributing factor to newborn and child health service inequality (26, 54, 55). Research conducted in Ethiopia reported that having four or more ANC visits was associated with a higher likelihood of receiving newborn care (56–58). However, currently, ANC service utilization is low, only four out of ten women have received four or more ANC visits in Ethiopia (6). According to the reports of previous studies, ANC service utilization was much lower among the women from economically disadvantaged groups (51, 59). The decomposition analysis in this study implies that the socioeconomic inequality in the postnatal health checks for newborns would reduce by 59%, if the ANC visits equally distributed among the women. Therefore, our findings highlight ANC visit is an important target variable for policy intervention. Efforts are required to improve four or more ANC visits in Ethiopia among economically disadvantaged women. Addressing inequality in ANC visits can significantly reduce the overall socioeconomic inequality postnatal health check for newborn.

The wealth status of the household was the second most contributing factor to the observed inequality. It explained about 34.43% of the overall inequality in postnatal health checks for newborns. This finding was similar to that in previous literature (54, 60). This could be women with higher wealth status can easily receive postnatal health checks for newborns anywhere at health facilities. In Ethiopia, maternal and newborn health care services are provided free of charge (free of direct medical costs) in public health facilities. However, women from lower socioeconomic groups, non-medical costs can impose a considerable financial burden. Non-medical costs such as transport and accommodation costs can deter women from obtaining a postnatal health check for their newborn.

This study also found that about 8.58% of the overall socioeconomic inequality in postnatal health checks for newborns was explained by the educational level of the mother. Several studies documented that the educational level of the mother was a significant contributing factor for socioeconomic inequality in child health services (26, 60–62). Possible explanations for this finding could be that educated women may be relatively empowered and have more autonomy than their poorer counterparts. Additionally, they have relatively better access to and use of health information. Furthermore, educated women can easily understand the benefits of a postnatal health check for newborns (63).

### Strengths and limitations

The strengths of this study include: to the best of our knowledge, this is the first study in Ethiopia to measure socioeconomic inequality in postnatal health checks for newborns using decomposition analysis. A nationally representative survey from the most recent DHS surveys was employed in the study. The findings of this study can help policymakers and planners to design evidence-based interventions. However, our study has some limitations. Since the DHS lacks direct measures of income, spending, or consumption, an asset-based wealth index was utilized in this study as a proxy for socioeconomic status. Second, because the study employed a cross-sectional survey, it was not possible to determine a causal relationship between the newborn’s determinant and the postnatal health check. Third, there was a time gap between the child’s birth and the interview period, as a result, there will be a possibility of recall bias.

## Conclusion

The results of the present study confirmed that there was a pro-rich inequality in postnatal health checks for newborns in Ethiopia. ANC visit, wealth status of the household, and educational status of the mother were the major contributing factors to the pro-rich socioeconomic inequalities of postnatal health checks for newborns. Therefore, to reduce the socioeconomic related health inequality in postnatal health checks for newborns, the government needs to improve ANC visits, implement strategies to access health service for economically disadvantaged groups, and increase educational attainment among women.

## Data availability statement

The original contributions presented in the study are included in the article/supplementary material, further inquiries can be directed to the corresponding author.

## Ethics statement

No ethical clearance was required because we used data from the DHS program. However, in order to access the data, requests for measure DHS and online registration were made. After obtaining authorization, the dataset was obtained from the DHS online archive (http://www.dhsprogram.com). All procedures were followed in compliance with the Helsinki Declaration.

## Author contributions

AH: Conceptualization, Data curation, Formal analysis, Investigation, Methodology, Project administration, Resources, Software, Validation, Visualization, Writing – original draft, Writing – review & editing. MT: Data curation, Formal analysis, Methodology, Resources, Software, Visualization, Writing – review & editing. KA: Data curation, Formal analysis, Methodology, Resources, Visualization, Writing – review & editing. YT: Data curation, Formal analysis, Investigation, Methodology, Resources, Software, Validation, Visualization, Writing – review & editing. AE: Formal analysis, Investigation, Methodology, Software, Validation, Visualization, Writing – review & editing. WN: Data curation, Formal analysis, Investigation, Methodology, Resources, Software, Validation, Visualization, Writing – review & editing. AW: Data curation, Formal analysis, Methodology, Resources, Software, Writing – review & editing. LY: Data curation, Formal analysis, Investigation, Methodology, Resources, Visualization, Writing – review & editing. MG: Data curation, Formal analysis, Methodology, Visualization, Writing – review & editing. NW: Data curation, Formal analysis, Investigation, Methodology, Resources, Software, Validation, Visualization, Writing – review & editing. AB: Data curation, Formal analysis, Investigation, Methodology, Writing – review & editing. LA: Investigation, Methodology, Resources, Validation, Writing – review & editing. HA: Data curation, Formal analysis, Methodology, Software, Validation, Writing – review & editing. DG: Formal analysis, Investigation, Methodology, Software, Visualization, Writing – review & editing. KD: Formal analysis, Investigation, Methodology, Software, Visualization, Writing – review & editing. MJ: Investigation, Methodology, Resources, Software, Supervision, Writing – review & editing.
